# Comparison of Bayesian and classical methods in the analysis of cluster randomized controlled trials with a binary outcome: The Community Hypertension Assessment Trial (CHAT)

**DOI:** 10.1186/1471-2288-9-37

**Published:** 2009-06-16

**Authors:** Jinhui Ma, Lehana Thabane, Janusz Kaczorowski, Larry Chambers, Lisa Dolovich, Tina Karwalajtys, Cheryl Levitt

**Affiliations:** 1 Department of Epidemiology and Biostatistics, McMaster University, Hamilton, Ontario, Canada; 2 Centre for Applied Health Research & Evaluation, University of British Columbia, Vancouver, British Columbia, Canada; 3 Department of Epidemiology and Community Medicine, University of Ottawa, Ottawa, Ontario, Canada; 4 Department of Family Medicine, McMaster University, Hamilton, Ontario, Canada

## Abstract

**Background:**

Cluster randomized trials (CRTs) are increasingly used to assess the effectiveness of interventions to improve health outcomes or prevent diseases. However, the efficiency and consistency of using different analytical methods in the analysis of binary outcome have received little attention. We described and compared various statistical approaches in the analysis of CRTs using the Community Hypertension Assessment Trial (CHAT) as an example. The CHAT study was a cluster randomized controlled trial aimed at investigating the effectiveness of pharmacy-based blood pressure clinics led by peer health educators, with feedback to family physicians (CHAT intervention) against Usual Practice model (Control), on the monitoring and management of BP among older adults.

**Methods:**

We compared three cluster-level and six individual-level statistical analysis methods in the analysis of binary outcomes from the CHAT study. The three cluster-level analysis methods were: i) un-weighted linear regression, ii) weighted linear regression, and iii) random-effects meta-regression. The six individual level analysis methods were: i) standard logistic regression, ii) robust standard errors approach, iii) generalized estimating equations, iv) random-effects meta-analytic approach, v) random-effects logistic regression, and vi) Bayesian random-effects regression. We also investigated the robustness of the estimates after the adjustment for the cluster and individual level covariates.

**Results:**

Among all the statistical methods assessed, the Bayesian random-effects logistic regression method yielded the widest 95% interval estimate for the odds ratio and consequently led to the most conservative conclusion. However, the results remained robust under all methods – showing sufficient evidence in support of the hypothesis of no effect for the CHAT intervention against Usual Practice control model for management of blood pressure among seniors in primary care. The individual-level standard logistic regression is the least appropriate method in the analysis of CRTs because it ignores the correlation of the outcomes for the individuals within the same cluster.

**Conclusion:**

We used data from the CHAT trial to compare different methods for analysing data from CRTs. Using different methods to analyse CRTs provides a good approach to assess the sensitivity of the results to enhance interpretation.

## Background

Cluster randomized trials (CRTs) are increasingly used in the assessment of the effectiveness of interventions to improve health outcomes or prevent diseases [1]. The units of randomization for such trials are groups or clusters such as family practices, families, hospitals, or entire communities rather than individuals themselves. CRT designs are used to evaluate the effectiveness of not only group interventions but also individual interventions where group-level effects are relevant. CRTs may also lead to substantially reduced statistical efficiency compared to trials that randomize the same number of individuals [2]. They may also produce selection bias since the allocation arm that the subject receives is often known in advance [3]. However, in practice, CRT designs have several attractive features that may outweigh these disadvantages. Cluster randomization minimizes the likelihood of contamination between the intervention and the control arms. In addition, the nature of the intervention itself may dictate its application as the optimal strategy [4].

The main consequence of a cluster design is that the outcomes for subjects within the same cluster can not be assumed to be independent. This is because the subjects within the same cluster are more likely to be similar to each other than those from different clusters. This leads to a reduction in statistical efficiency due to clustering, *i.e*. the design effect. The design effect is a function of the variance inflation factor (VIF), given by 1 + ( - 1)*ρ*, where  denotes the average cluster size and *ρ *is a measure of intra-cluster correlation – interpretable as the correlation between any two responses in the same cluster [2,5]. Considering the two components of the variation in the outcome, between-cluster and within-cluster variations, *ρ *may also be interpreted as the proportion of overall variation in outcome that can be accounted for by the between-cluster variation.

These principles are well established in the design of CRTs, especially when there are implications for the sample size planning. In statistical analysis, it has long been recognized that ignoring the clustering effect will increase the chance of obtaining statistically significant but spurious findings [6]. Although many papers have compared analytical methods for CRTs with binary outcomes over the last decade, none have investigated the Bayesian model in the analysis of CRT in detail. In particular, comparison of random-effects meta analytic approach with other methods for the analysis of matched pair CRTs [7] has not been done. In this paper, we compare various statistical approaches in the analysis of CRTs using the Community Hypertension Assessment Trial (CHAT) as an example. The CHAT study is a multi-centre randomized controlled trial using blocked stratified, matched-pair cluster randomization. We also explored in much detail the application of the Bayesian random-effects model in the analysis of CRTs. In particular, we investigated the impact of different prior distributions on the estimate of the treatment effect.

## Methods

### Overview of the CHAT study

The CHAT study was a cluster randomized controlled trial aimed at investigating the effectiveness of pharmacy-based blood pressure (BP) clinics led by peer health educators, with feedback to family physicians (FP) on the monitoring and management of BP among older adults [8]. The participants of the trial included the FP practices, patients, pharmacies and peer health educators. Eligible FPs were non-academic, full-time practitioners with regular family practices in terms of size and case-mix, and were able to provide an electronic roster that included a mailing address of their patients 65 years and older. FPs who worked in walk-in clinics or emergency departments, were about to retire or worked part-time, had fewer than 50 patients 65 years or older, or had a specialized practice profile were excluded from the study. Eligible patients were 65 years or older at the beginning of the study, considered by their FPs to be regular patients, community-dwelling and able to leave their homes to attend the community-based pharmacy sessions. To ensure that the results would be generalizable to patients in other FP practices, the trial had very few exclusion criteria for patients.

The study design was a multi-centre randomized controlled trial using blocked stratified, matched-pair cluster randomization. Family practices were the unit of randomization. Eligible practices were stratified according to (1) the median number of patients in the practice with adequate BP control and (2) the median number of patients aged 65 years and older, and matched according to centers. The trial started in 2003 with 28 FPs practising in Ottawa and Hamilton randomly selected from the eligible FPs. Fourteen were randomly allocated to the intervention (pharmacy BP clinics) and 14 to the control group (no BP clinics offered). Fifty-five eligible patients were randomly selected from each FP roster. Therefore, 1540 patients participated in the study.

All eligible patients in both the intervention or control group got usual health service at their FP's office. Patients in the practices allocated to the intervention group were invited to visit the community BP clinics. Peer health educators assisted patients to measure their BP and record their readings on a form that also asked about cardiovascular risk factors. Research nurses, assisted by the FP office staff, conducted the baseline and end-of-trial (12 months after the randomization) audits of the health records of the 1540 patients (55 per practice) who participated in the study. These data were collected to determine the effect of the intervention.

### Outcomes

The primary outcome of the CHAT study was a binary outcome with "1" indicating the patient's BP was controlled at the end of the trial and "0" otherwise. We defined that the patient's BP was controlled as follows:

• if the BP reading was available in the patient's chart at the end of the trial and the systolic BP ≤ 140 mmHg and diastolic BP ≤ 90 mmHg for patient without diabetes or target organ damage, or

• the systolic BP ≤ 130 mmHg and diastolic BP ≤ 80 mmHg for patient with diabetes or target organ damage.

Secondary outcomes of the CHAT study included 'BP monitored', frequency of BP monitoring, systolic BP reading, and diastolic BP reading. The analyses presented in this paper are based on the primary outcome only. The analysis of secondary outcomes will be the subject of another paper reporting the trial results.

### Statistical methods

The analysis of CRTs may be based on the analysis of aggregated data from each cluster or based on individual level data, which correspond to the cluster-level and the individual-level analysis methods, respectively. The adjustment for individual-level covariates may be applied only for the individual level analysis. While the adjustment for the cluster-level covariates may be applied for both the cluster-level and the individual-level analysis. In this paper, the random-effects meta-regression method was performed using STATA Version 8.2 (College Station, TX). Other standard analyses were performed using SAS Version 9.0 (Cary, NC). The Bayesian analysis was performed using WinBugs Version 1.4. The results from classical analyses for binary outcomes are reported as odds ratio (OR) and corresponding 95% confidence interval (CI). The results from the Bayesian method are reported as posterior estimate and corresponding 95% credible interval (CrI). CrIs are the Bayesian analog of confidence intervals. The reporting of the results follows the CONSORT (Consolidated Standards of Reporting Trials) statement guidelines for reporting cluster-randomized trials [9] and ROBUST guideline [10] for reporting Bayesian analysis.

### Cluster-level analyses methods

Following Peters *et al *[11], we assume that the number of patients in cluster/FP *i *(*i *= 1 to 28) with BP controlled and the total number of patients in the cluster/FP are denoted by *r*_*i *_and *n*_*i *_respectively. For the FP *i*, the log odds of number of patients with BP controlled is estimated as

and its variance is

#### Un-weighted linear regression

Considering log odds for each cluster as the dependent variable, the un-weighted linear regression model [12] can be expressed as:

where *x*_*i *_denotes the vector of covariates (intervention groups and centers), *β *represents the effect of the covariates in the log odds scale, and *u*_*i *_represents the cluster level random effect. The *u*_*i *_here is assumed to follow normal distribution with a zero mean and a constant variance. In this method, each cluster/FP is given equal weight when estimating the regression coefficient *β*. We implemented this model using SAS *proc glm*.

#### Weighted linear regression

The weighted linear regression method [12] has the same model expression as the un-weighted linear regression method. It treats the log odds estimated from each cluster as the outcome, and treatment group as one of the explanatory variables. The weight was defined as the inverse variance of the log odds, i.e. *w*_*i *_= 1/var_*i *_for FP *i*. Compared to the un-weighted linear regression – in which all cluster estimates are weighed equally – the weighted linear regression gave clusters with higher precision more weight, and therefore more contribution in estimating the treatment effect. We implemented this model using SAS *proc glm*.

#### Random-effects meta-regression

The random-effects meta-regression model [13] is similar to the un-weighted linear regression model:

However, the *u*_*i *_here is assumed to follow a normal distribution on the log odds scale with a zero mean and an uncertain variance, which represents the between cluster variance and can be estimated when fitting the model. We implemented this model using *STATA metareg*.

### Individual-level analyses

We used six individual-level statistical methods that extend the standard logistic regression methods by adding specific strategies to handle the clustering of the data, and therefore are valid for analyzing clustering data.

#### Standard logistic regression

The standard logistic regression model [14,15] can be expressed as:

where *x*_*ij *_denotes the vector of covariates (BP controlled at baseline, intervention groups etc.) for patient *j *in the cluster/FP *i*; *y*_*ij *_is the binary outcome indicating if the BP is controlled for patient *j *in the cluster/FP *i*; and *π*_*ij *_= Pr(*y*_*ij *_= 1|*x*_*ij*_).

The standard logistic model assumes that data from different patients are independent. Since this assumption is not valid for the correlated data, it is not valid for analyzing cluster randomized trials. We implemented this model using SAS *proc genmod*.

#### Robust standard errors

Like the standard logistic regression, the robust standard error method [14,16] gives the same estimates since both of them assume independent data to get the estimate of the treatment effect. However, in the robust standard errors method, the standard errors for all the estimates are obtained using 'Huber sandwich estimator' which can be used to estimate the variance of the maximum likelihood estimate when the underlying model is incorrect or the model assumption is wrong [17]. It is often used for clustered data [18]. We implemented this model using SAS *proc genmod*.

#### Generalized estimating equations

The generalized estimating equations (GEE) [14,19,20] method permits the specification of a working correlation matrix that accounts for the form of within-cluster correlation of the outcomes. In the analysis of CRTs, we generally assume that there is no logical ordering for individuals within a cluster, *i.e. *the individuals within the same cluster are equally correlated. In this case, an exchangeable correlation matrix should be used. We implemented this model using SAS *proc genmod*.

Though the sandwich standard error estimator is consistent even when the underlying model is specified incorrectly, it tends to underestimate the standard error of the regression coefficient when the number of clusters is not large enough [21,22]. Furthermore, the estimate of standard error is highly variable when the number of clusters is too small. In this paper, we employed two methods proposed by Ukoumunne [23] to correct this bias. Both methods can be used when there are equal numbers of clusters in each arm and no covariate adjustment. In the first method – modified GEE (1), the bias of the sandwich standard error is corrected by multiplying it by , where *J *is the number of clusters in each arm. In the second method – modified GEE (2), the increased variability of the sandwich standard error estimator was accounted for by building the confidence interval for the treatment effect based on the quantiles from the *t*-distribution with 2(*J*-1) degree of freedom.

#### Random-effects meta-analytic approach

This method is appropriate only for CRTs with matched pair design [2]. If we assume that the data from each paired cluster are arising from a meta-analysis of independent randomized controlled clinical trials, then we can apply the traditional random-effects meta-analysis method to pool the results from all the pairs [13]. The random-effects meta-analytic approach for analysing CRTs consists of two steps. First, the treatment effect is estimated for each paired cluster. Second, the overall treatment estimator is calculated as a weighted average of the paired cluster estimates, where weights are the inverse of the estimated variances of treatment effects of the paired clusters. We implemented this model using SAS *proc genmod *and *proc mixed*.

#### Random-effects logistic regression

The random-effects logistic regression [15,19] is a special kind of hierarchical linear model. Compared to the standard logistic regression, the random-effects logistic regression includes a cluster-level random effect in the model which is assumed to follow a normal distribution with a zero mean and an unknown variance *τ*^2 ^(the between-cluster variance); *τ*^2 ^is estimated in the regression. By allowing for over-dispersion parameter to be estimated, we adopted the estimating algorithm of pseudo-likelihood function of Wolfinger/O'Connell 1993 [24]. Compared to the Bayesian model, the CI for the treatment effect from this method is narrower since it is based on estimated constant variance components without allowance for uncertainty [25]. In practice, it may be difficult to assess the validity of the model assumption that the cluster-level random-effects follow a normal distribution. We implemented this model using SAS *macro glimmix*.

#### Bayesian random-effects regression

The Bayesian random-effects regression model [26] has the same format as the traditional random-effects logistic regression. However it is based on different assumptions to the variance of the cluster level random effect. The Bayesian approach assumes the variance of the random effect *τ*^2 ^as an unknown parameter while the traditional regression approach assumes it as a constant. In the Bayesian approach, the uncertainty of *τ*^2 ^is taken into account by assuming a prior distribution which presents the researcher's pre-belief or external information to *τ*^2^. The observed data are presented as a likelihood function, which is used to update the researcher's pre-belief and then obtain the final results. The final results are presented as the posterior distribution.

When applying the Bayesian model, it is essential to state in advance the source and structure of the prior distributions that are proposed for the principal analysis [27,28]. In our Bayesian analysis, we assumed the non-informative uniform prior distribution with lower and upper bounds as 0 and 10 respectively to minimize the influence of the researcher's pre-belief or external information on the observed data. Consequently, the result from the Bayesian approach should be comparable to the results from the classical statistical methods. We also assumed that the prior distribution for all the coefficients follows a normal distribution with a mean of zero and precision 1.0E-6. The total number of iterations to obtain the posterior distribution for each end point is 500,000, the burned-in number is 10,000, and the seed is 314159. The non-convergence of the Markov Chain is evaluated by examining the estimated Monte Carlo error for posterior distributions and a dynamic trace plots, times series plots, density plots and autocorrelation plots.

### Impact of priors for Bayesian analysis

Even though the researcher's subjective pre-beliefs, which are expressed as prior distribution functions, can be updated by the likelihood function of the observed data, misspecification of priors has an impact on the posterior in some cases. To verify the robustness of the results from the Bayesian random-effects logistic regression, we evaluated the impact of different prior distributions of the variance parameter in the analysis of the primary outcome, BP controlled, without adjustment for any covariates.

The commonly used priors for the variance parameter are uniform (non-informative prior) and inverse gamma (non-informative and conjugate prior) [29]. The estimates of treatment effect when assuming different prior distributions were quite consistent based on the results presented in Table 1. For uniform priors, the estimates and the 95% CIs were similar when the upper bound of the uniform distribution was greater than or equal to 5. For the inverse-gamma prior, Gelman [29] pointed out that when *τ*^2 ^is close to zero, the results may be sensitive to different choices of the parameter *ε*. Since *τ*^2 ^is approximately 0.5 (estimated from random-effects meta regression, random-effects logistic regression and Bayesian approaches), which is not close to zero, our results are stable when using inverse-gamma prior with different choices of the parameter *ε*.

**Table 1:** Comparison of the Impact of Different Priors on Bayesian Model

Prior		Outcome: BP controlled (unadjusted for covariates)	
Type of Prior	Prior distribution	Odds Ratio	95% CI

	Uniform (0, 1)	1.11	(0.64 1.92)
	Uniform (0, 5)	1.09	(0.61 1.94)
Non-informative	Uniform (0, 10)	1.09	(0.61 1.94)
	Uniform (0, 50)	1.09	(0.61 1.94)
	Uniform (0, 100)	1.09	(0.61 1.94)

Non-informative and Conjugate	IGamma (0.001, 0.001)	1.11	(0.63 1.94)
	IGamma (0.01, 0.01)	1.11	(0.63 1.95)
	IGamma (0,1, 0.1)	1.12	(0.64 1.95)

CI = confidence interval; BP = Blood pressure; Igamma = Inverse Gamma

## Results

Since the data collection was based on chart review, there were very few missing values for the CHAT study. Demographic information and health conditions were balanced between the two study arms at baseline. Of the 1540 patients who were included, there were 41% (319/770) male patients in the control group and 44% (339/769) male patients in the intervention group. At the beginning of the trial, the mean age of the patients was 74.36 with a standard deviation (SD) of 6.22 in the control group, and 74.16 with SD of 6.14 in the intervention group. In the intervention and control group, 55% (425/770) and 55% (420/770) of patients had BP controlled at baseline; 57% (437/770) and 53% (409/770) of patients had BP controlled at the end of the trial.

In analyzing the binary primary outcomes of the CHAT trial (BP controlled), the results from different statistical methods were different. However, the estimates obtained from all of the nine methods showed that there were no significant differences in improving the patients' BP between the intervention and the control groups.

**Figure 1:**
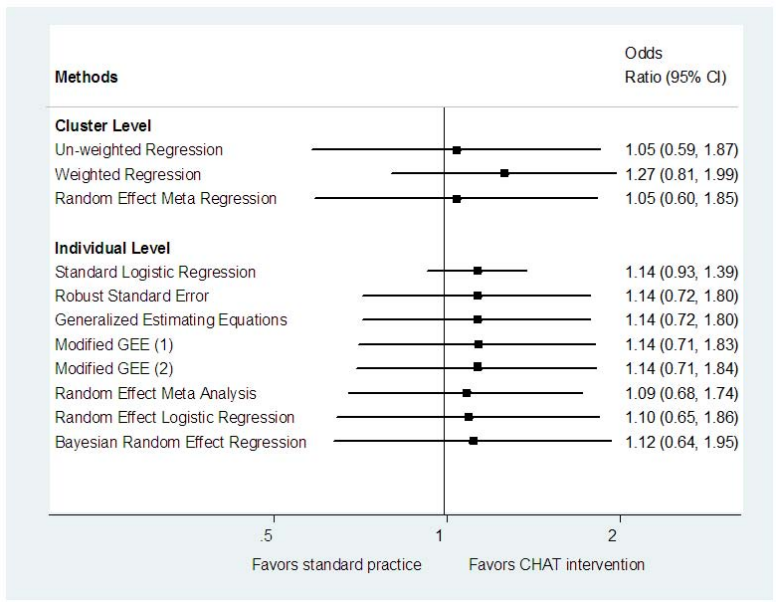
**Forest Plot: Comparison of Methods without Adjustment for Covariates**.

For the cluster-level methods, we compared the odds ratios and 95% confidence interval with and without adjustment for the stratifying variable, 'centre' (Hamilton or Ottawa). The variable 'centre' is not significant at *α *= 0.05 in predicting if the patients' BPs were controlled at the end of the trial. When adjusting for covariate 'centre', the treatment effects were slightly different and the 95% confidence intervals for the treatment effects were narrower. For individual-level methods, we compared the results of the analysis with and without adjustment for patients' characteristics at baseline. These baseline characteristics included diabetes, heart disease, and whether or not the BP of the patient was controlled at baseline. All of these covariates were significantly associated with the outcome at level *α *= 0.05. When we included some patients' baseline information as the covariates in the models, the odds ratios of the treatment effect changed slightly and the 95% confidence intervals tended to be much narrower compared to estimates without adjustment for any covariate. The intra-cluster correlation coefficient (ICC) reduced from 0.077 to 0.054 after adjusting for covariates. The 95% confidence intervals for the treatment effect from the two modified GEE models became slightly wider after the bias of sandwich standard error estimator was corrected, but our conclusions remained robust. The comparison of the results from different statistical methods is presented in Table 2 and Figure 1.

**Table 2:** Comparison of Nine Methods with and without Adjustment for Covariates

Unit of Analysis	Method of Analysis	Unadjusted for Covariates		Adjusted for Covariates	
		
		OR	95% CI	OR	95% CI
Cluster	Un-weighted Regression	1.05	(0.59 1.87)	1.05	(0.60 1.84)
	Weighted Regression	1.27	(0.81 1.99)	1.27	(0.82 1.96)
	Random-effects Meta Regression	1.05	(0.60 1.85)	1.05	(0.61 1.82)

Individual	Standard Logistic Regression	1.14	(0.93 1.39)	1.17	(0.95 1.44)
	Robust Standard Error	1.14	(0.72 1.80)	1.17	(0.79 1.73)
	Generalized Estimating Equations **	1.14	(0.72 1.80)	1.15	(0.76 1.72)
	Modified GEE (1) ***	1.14	(0.71 1.83)		
	Modified GEE (2) ****	1.14	(0.71 1.84)		
	Random-effects Meta Analysis	1.09	(0.68 1.74)	1.12	(0.73 1.70)
	Random-effects Logistic Regression	1.10	(0.65 1.86)	1.13	(0.71 1.80)
	Bayesian Random-effects Regression	1.12	(0.64 1.95)	1.13	(0.68 1.87)

OR = odds ratio; CI = confidence interval

* For the cluster level analysis, include 'center' (*i.e. *Hamilton and Ottawa) as the covariate; for the individual level analysis, include 'diabetes at baseline', 'heart disease at baseline', and 'BP controlled at baseline' as the covariates.

** The intra-cluster correlation coefficient (ICC) estimated from GEE are 0.077 and 0.054 when unadjusted for covariates and adjusted for covariates respectively.

*** The confidence interval was calculated based on the corrected standard error which was equal to the sandwich standard error estimator multiply by , where *J *is the number of clusters in each arm.

**** The Confidence interval was calculated based on the quantiles from the *t*-distribution with 2(*J*-1) degrees of freedom instead of quantiles from the standard normal distribution.

## Discussion

### Summary of Key Findings

We applied three cluster-level and five individual-level approaches to analyse results of the CHAT study. We also employed two methods to correct the bias of the sandwich standard error estimator from the GEE model. Among all the analytic approaches, only the individual-level standard logistic regression was inappropriate since it does not account for the between-cluster variation. This is because it tends to underestimate the standard error of the treatment effect and its *p*-value. Correspondingly, this method might exaggerate the treatment effect. All the other methods handle the clustering by different techniques, and therefore were appropriate. All but the weighted regression method yielded similar point estimates of the treatment effect. This is not surprising since the weighted regression method can potentially affect the location of the estimate as well as the precision. The Bayesian random-effects logistic regression yielded the widest confidence interval. This was due to the fact that the Bayesian random-effects logistic regression incorporates the uncertainty of all parameters. The 95% confidence intervals for the treatment effect from the two modified GEE models are slightly wider than that from the GEE model. Adjusting for important covariates that are correlated with the outcome increased the precision and reduced the ICC. This is consistent with the finding from Campbell for the analysis of cluster trials in family medicine with a continuous outcome [30]. By adjusting for important covariates, we are able to control for the effect of imbalances in baseline risk factors and reduce unexplained variation. In general, it is important to note that for logistic regression, the population averaged model (fitted using GEE) and the cluster specific method (modelled by random effects models) are in fact estimating different population models. This is covered in detail by Campbell [31] and was first discussed by Neuhaus and Jewell [32]. Thus, we would not expect the estimates for the GEE and the random-effects logistic regression to be exactly the same. However, they are related through the ICC [31]. In our case, the estimates from the two models are similar since the ICC in the CHAT study is relatively small.

### Sensitivity analysis and simulation study

Several sensitivity analyses can be considered for CRTs. First, since different methods yield different results, and very few methodological studies provide guidance on determining which method is the best, comparing the results from different methods might help researchers to draw a safer conclusion, though the marginal odds ratio estimated by the GEE and the conditional odds ratio estimated from random-effect models may be interpreted differently [33]. Second, sensitivity analysis can be used to investigate the sensitivity of the conclusions to different model assumptions. For example, in the random-effects model, we assume that the cluster-level random effects follow a normal distribution on the log odds scale. However, a sensitivity analysis can be carried out by allowing empirical investigation on the distribution of the random effects. Finally, a sensitivity analysis can also indicate which parameter values are reasonable to use in the model.

The Bayesian analysis incorporates different sources of information in the model. However, a disadvantage of this technique is that the results of the analysis are dependent on the choice of prior distributions. We performed more analyses to assess the sensitivity of the results to different prior distributions representing weak information (*i.e. *non-informative prior) relative to the trial data, and the results remained robust.

A simulation study by Austin [34] suggested that the statistical power of GEE is the highest among t-test, Wilcoxon rank sum test, permutation test, adjusted chi-square test and logistic random-effects model for the analysis of CRTs. However, researchers should be cautioned about the limitations of the GEE method. First, when the number of clusters is small, the estimate of variance produced under GEE could be biased [21,22], particularly if the number of clusters is less than 20 [35]. In this case, correction for the bias would be necessary. Second, the research on the goodness-of-fit tests to the GEE application still faces some challenges [36]. Third, Ukoumunne *et al *[23] compared the accuracy of the estimation and the confidence interval coverage from three cluster-level methods – the un-weighted cluster-level mean difference, weighted cluster-level mean difference and cluster-level random-effects linear regression – and the GEE model in the analysis of binary outcome from a CRT. Their results showed that the cluster-level methods performed well for trials with sufficiently large number of subjects in each cluster and a small ICC. The GEE model led to some bias of the sandwich standard error estimator when the number of clusters are relatively few. However, this bias could be corrected by multiplying the sandwich standard error by , where *J *is the number of clusters in each arm, or by building the confidence interval for the treatment effect based on the quantiles from the *t*-distribution with 2(*J*-1) degree of freedom. With these corrections, the GEE was found to have good properties and would be generally preferred in practice over the cluster-level methods since both cluster-level and individual-level confounders can be adjusted for.

## Conclusion

We used data from the CHAT trial to compare different methods for analysing data from CRTs. Among all the statistical methods, Bayesian analysis gives us the largest standard error for the treatment effect and the widest 95% CI and therefore provides the most conservative evidence to the researchers. However, the results remained robust under all methods – showing sufficient evidence in support of the hypothesis of no effect for the CHAT intervention against Usual Practice control model for management of blood pressure among seniors in primary care. Our analysis reinforces the importance of building sensitivity analyses to support primary analysis in analysis of trial data so as to assess impact of different model assumptions on results. Nonetheless, we cannot infer from these analyses which method is superior in the analysis of CRTs with binary outcomes. Further research based on simulation studies is required to provide better insights into the comparability of the methods in terms of statistical power for designing CRTs.

## Competing interests

The authors declare that they have no competing interests.

## Authors' contributions

JM and LT conceived the study. LT, JK, LC, LD, TK and CL participated in the design and implementation of the CHAT study. Data cleaning was done by TK and JM. JM conducted the data analysis and wrote the initial draft of the manuscript. Results of the data analysis were interpreted by JM and LT. All authors reviewed and revised the draft version of the manuscript. All authors read and approved the final version of the manuscript.

## Pre-publication history

The pre-publication history for this paper can be accessed here:

http://www.biomedcentral.com/1471-2288/9/37/prepub
